# Difficulties and Feelings Experienced by Undergraduate Dental Students When Caring for Patients

**DOI:** 10.1002/jdd.70005

**Published:** 2025-08-29

**Authors:** Bruna B. Rodrigues, Ana L. Santos‐Sousa, Tamara F. Castro, Vitor B. Valente, Glauco I. Miyahara, Daniel G. Bernabé

**Affiliations:** ^1^ Psychosomatic Research Center Oral Oncology Center School of Dentistry São Paulo State University (UNESP) Araçatuba São Paulo Brazil; ^2^ Department of Diagnosis and Surgery School of Dentistry São Paulo State University (UNESP) Araçatuba São Paulo Brazil

**Keywords:** clinical examination, dental students, feelings, interpersonal relationships, oral medicine, patient care

## Abstract

**Purpose:**

Dealing with the patient's illness process is part of the academic trajectory of healthcare students. The relationship between patients and their healthcare professionals can be complex and challenging, especially for undergraduate dental students. The purpose of this study was to evaluate the perceptions, feelings, and difficulties of dental students triggered when caring for patients during their undergraduate course.

**Methods:**

Eighty‐six undergraduate dental students responded to a semi‐structured questionnaire about their experiences with patient care in the oral medicine clinic.

**Results:**

Forty‐two students (48.8%) felt uncomfortable with the patients’ anamnesis questions. Among these, the inquiries about sexually transmitted infections (STIs) were the most discomforting topic to address with the patient (65%), followed by drug abuse (35%) and sexual behavior (15%). According to the students, the most difficult topic to discuss with the patient was a negative diagnosis (40%), followed by STIs and sexual behavior (25.3%), and physical and sexual violence (20%). During patient care, the main challenging issue was dealing with uncooperative patients, reported by 29 students (36.7%). About the challenges faced during patient interviews, the majority of the students reported feeling bad when the patient shared a negative life story (90.7%) or when they described a traumatic life event (83.7%). A portion of the students (26.7%) reported that this discomfort in hearing a patient's negative life story can negatively impact the care they provide to the patient.

**Conclusion:**

Dental students experience evident difficulties in clinical practice with patients in the oral medicine clinic, especially in relation to some themes addressed during the anamnesis. Knowledge of these difficulties is essential to develop new learning and teaching strategies and improve the students’ professional training during their dental undergraduate course.

## 1 Introduction

The interpersonal relationship between healthcare providers and their patients can be challenging and complex [1]. The complexity of human relationships is intimately related to the success of professional performance. The professional–patient relationship (PPR) goes beyond technical knowledge. During patient care, the professional is faced with the patient's emotions, feelings, and anxieties that may arise during the diagnosis and treatment processes [2]. Patients' higher trust in their healthcare professionals is related to higher patient satisfaction and health‐related quality of life [3]. Due to the high anxiety and dental fear among patients in oral healthcare, this confidence must be enhanced in the relationship between patients and their dentists [4]. However, considering the challenge of dealing with patient emotions and other difficulties, 60% of dental professionals consider dentistry to be more stressful than other professions [5].

A healthy and harmonious relationship between professionals and patients is necessary for successful treatment and involves empathy and compassion. Having empathy and considering the patient's emotions can contribute to building an emotional bond that is favorable to dental treatment [6]. Despite the importance of dentistry's interaction with patients’ feelings and insecurities, there are few investigations in this field that can improve this discussion and provide alternatives to manage the challenges of PPR in dentistry [7]. This can result in insufficient professional training to develop dentists’ abilities to manage the adversities of professional practice and lead to undesirable consequences. This gap in dental education can lead to negative professional consequences, including mental illness and professional immaturity in dealing with the real‐world challenges [1, 5, 7]. Insufficient training and education for the real difficulties of the professional field can also lead to professional disappointment and career abandonment [7]. Bringing students closer to real professional experiences throughout their undergraduate course and exposing them to challenges can provide several benefits for their professional future [7].

Caring for patients in physical and emotional suffering requires high levels of emotional control. Healthcare students who are not sufficiently prepared to deal with patient distress may easily develop negative emotional symptoms. In contrast, healthcare students who already have experience in dealing with patients’ emotions and insecurities are better prepared to manage adverse PPR situations [8, 9, 10]. Dental students can identify their own life histories and previous experiences in the patients’ feelings. This projection can trigger students’ negative emotions and can be related to difficulties in continuing the patient's care. In this context, addressing a patient's suffering situation can remind the professional of personal traumatic experiences and evoke psychological distress in the professional [11, 12]. Furthermore, healthcare students with impaired mental health can be more impacted by patients’ life stories and suffering, and, consequently, can present impairment in their care provision [13, 14, 15, 16, 17].

Identifying the main difficulties that dental students face during patient care can guide educators to the topics that need to be better addressed during the dental student's professional training. Although there is extensive research on this topic among other healthcare providers, only a few investigations have been conducted among dental students [11, 12]. Therefore, this research aimed to evaluate and address the difficulties and feelings of dental students triggered when caring for patients during oral medicine clinical practice.

## 2 Volunteers and Methods

### 2.1 Volunteers

Eighty‐six undergraduate dental students were recruited during the second semester of the oral medicine discipline of the Araçatuba Dental School, São Paulo State University (UNESP), Araçatuba, São Paulo, Brazil. The discipline of oral medicine is offered to undergraduate students throughout the entire third year of the dentistry course and represents the students’ first exposure to comprehensive clinical training, integrating theory and practice for complete patient assessment. Before taking this discipline, the students had foundational disciplines that only included bacterial plaque identification, oral hygiene evaluation, and orofacial radiography. These disciplines did not include full clinical patient examination training. To answer the questionnaire, the students were gathered in a room and informed about the research objectives. All students agreed to participate in the study, signed the informed consent form (ICF), and completed the questionnaire individually and self‐responsively. This research was approved by the Research Ethics Committee for Human Subjects (CAAE: 71276123.0.0000.5420; Protocol Number: 6.206.066). Informed consent was obtained from all volunteers participating in the study.

### 2.2 Semi‐Structured Questionnaire and Study Variables

The study data were obtained through a semi‐structured questionnaire elaborated by the researchers to evaluate the students' experience during the patients’ assistance in the discipline of oral medicine. Data collected included demographic, medical, and behavioral variables. Demographic variables were age, sex (male or female), religion, and socioeconomic status. Medical variables included alcoholism, smoking, psychiatric disorders, diseases under treatment, use of medications, and family members’ illnesses. The difficulties and feelings experienced by students during the clinical examination of the patient were assessed by the following questions: “In the context of clinical anamnesis, do you feel uncomfortable asking the patient any questions? If yes, indicate which question and the reason why it makes you uncomfortable”; “Do you have difficulty discussing any specific topics with patients? If yes, indicate which topic and the reason”; “During the whole patients’ care, does any topic make you uncomfortable? If yes, indicate which topic and the reason”; “Do you feel bad when the patient tells you a negative life story?”; “Do you feel bad when the patient reports traumatic emotional events that occurred in their life?”; “Do you feel discomfort when the patient tells a negative life story similar to yours?”; “When the patient tells you a negative life story, does this impact the care you provided?”, that could be answered with “Yes, it negatively impacts the care provided” or “No, it does not impact the care provided”; “Have you had a negative judgment towards patients when they answered some question in the interview? If yes, indicate which question topic whose answer generated the negative judgment” and “Do you have a negative judgment or annoyance regarding the patient's difficulty in understanding certain questions?”

### 2.3 Discourse of the Collective Subject Analysis

The data from the open‐ended questions were analyzed using the Discourse of the Collective Subject (DCS). This qualitative technique analyzes collective dialog threads, extracting individual expressions, keywords, and similar statements made by participants and synthesizing them into a unified discourse [10].

### 2.4 Statistical Analysis

Frequency analysis of all variables was performed using Microsoft Office Excel 2019. The data derived from the closed questions were presented as the frequency of positive or negative answers. For the open questions, the frequency of the central idea derived from the DCS analysis corresponded to the number of students who answered “Yes” to the respective question.

## 3 Results

### 3.1 Dental Students’ Epidemiological Profile

A total of 86 undergraduate dental students were included in the study. The sample comprised 63 women (73.2%) and 23 men (26.8%), with a mean age of 21.8 years (SD = 1.34). Sixty‐four students (74.5%) reported having a religious affiliation, and most of the volunteers reported household incomes consistent with middle socioeconomic status (89.5%). Regarding the use of legal substances, 66.3% of the sample declared some level of alcohol consumption, while 17.4% reported tobacco use. Twenty‐one students were undergoing treatment for some medical condition at the time of the interview. For psychological disorders, 11.6% reported diagnosed and treated anxiety, and 8.1% reported diagnosed and treated depression (Table 1).

**TABLE 1:** Sociodemographic characteristics of students.

**Variables**	**Students**
	**No. (%)**
**Gender**	
Female	63 (73.2)
Male	23 (26.8)
**Mean age (years, SD)**	21.8 (± 1.34)
**Religion**	
Some religion	64 (74.5)
Without religion	22 (25.5)
**Socioeconomic status**	
High‐income	8 (9.3)
Middle‐income	77 (89.5)
Low‐income	1 (1.2)
**Alcohol consumption**	57 (66.3)
Smoker	15 (17.4)
Illnesses under treatment	21 (24.4)
**Psychiatric disorders**	
Anxiety	10 (11.6)
Depression	7 (8.1)

### 3.2 Uncomfortable Topics in Patient Communication

Forty‐two students (48.8%) reported discomfort when addressing a specific topic during patient anamnesis. The topic causing the most discomfort was Sexually Transmitted Infections (STIs) (65.0%), followed by drug abuse (35.0%), sexual behavior (15.0%), and questions about the patient's race and personal life (7.5%). During overall patient care, 92.9% of the students reported experiencing discomfort. The primary source of discomfort was dealing with uncooperative patients (36.7%), followed by communicating negative diagnoses (15.2%) and discussing patients’ personal lives (11.4%). Thirty‐six students (41.9%) reported avoiding certain topics during patient conversations. The most challenging topics to discuss were negative diagnosis (40.0%), STIs and sexual behavior (25.3%), and situations involving physical or sexual violence (20.0%) (Table 2).

**TABLE 2:** Topics that students have difficulty asking or discussing with the patient.

Questions/Data	**Students**
	**No. (%)**
**I** **n the context of clinical anamnesis, do you feel uncomfortable asking the patient any questions** **?** (*n* = 86)	
Yes	42 (48.8)
No	44 (51.2)
**If yes, indicate which question and the reason why it makes you uncomfortable** (*n* = 40).	
Sexually transmitted infections	26 (65.0)
Drug abuse	14 (35.0)
Sexual behavior	6 (15.0)
Race	3 (7.5)
Patients’ personal life	3 (7.5)
**Do you have difficulty discussing any specific topics with patients?** (*n* = 85)^a^	
Yes	75 (88.2)
No	10 (11.8)
**If yes, indicate which topic and the reason** (*n* = 75)^b^	
Negative diagnoses	30 (40.0)
Physical or sexual violence	15 (20.0)
Sad topics	13 (17.3)
Sexually transmitted diseases	12 (16)
Death	10 (13.3)
Sexual behavior	7 (9.3)
Drug abuse	4 (5.3)
Topics the students do not know	2 (2.7)
Students’ personal life	2 (2.7)
**During the whole patients’ care, does any topic make you uncomfortable?** (*n* = 85)^a^	
Yes	79 (92.9)
No	6 (7.1)
**If yes, indicate which topic and the reason** (*n* = 79)^a^, ^b^	
Uncooperative patients	29 (36.7)
Negative diagnoses	12 (15.2)
Patients’ personal life	9 (11.4)
Physical pain	8 (10.1)
Failure to communicate with the patient	7 (8.9)
Sexually transmitted infections and sexual behavior	4 (5.1)
Long duration of service time	5 (6.3)
Use of substances	3 (3.8)

^a^ Variables with missing data.

^b^ Questions interpreted by Discourse of the Collective Subject (DCS).

### 3.3 Students’ Discomfort With Patients' Life Stories

When asked about their reactions to patients sharing negative life stories or traumatic events, 78 (90.7%) and 72 students (83.7%) reported emotional discomfort, respectively. Sixty‐nine students (80.2%) expressed discomfort when patients shared negative life stories with which they personally identified. However, most students (73.3%) reported that the patients’ negative life stories do not affect the quality of care they provided (Table 3).

**TABLE 3:** Students' difficulties during patient interviews.

**Questions/Data**	**Students**
	**No. (%)**
**Do you feel bad when the patient tells you a negative life story?** (*n* = 86)	
Yes	78 (90.7)
No	8 (9.3)
**Do you feel bad when the patient reports traumatic emotional events that occurred in their life?** (*n* = 86)	
Yes	72 (83.7)
No	14 (16.3)
**Do you feel discomfort when the patient tells a negative life story similar to yours?** (*n* = 86)	
Yes	69 (80.2)
No	17 (19.8)
**When the patient tells you a negative life story, does this impact on the care you provided?** (*n* = 86)	
Yes, it negatively impacts the care provided	23 (26.7)
No, it does not impact the care provided	63 (73.3)

### 3.4 Judgments Toward Patient

Twenty‐nine students (33.7%) admitted to having expressed a negative judgment about patients during clinical interviews. Participants reported this judgment most frequently when they perceived patients' neglect of their own health (50.0%), a previous STI diagnosis (23.1%), and a history of drug use (23.1%) or alcohol abuse (15.4%). In addition, 24 students (27.9%) reported frustration with patients’ difficulty understanding certain interview questions (Table 4).

**TABLE 4:** Students' judgment towards the patient during clinical interviews.

**Questions/Data**	**Students**
	**No. (%)**
**Have you had a negative judgment towards patients when they answered some question in the interview?** (*n* = 86)	
Yes	29 (33.7)
No	57 (66.3)
**If yes, indicate which question topic whose answer generated the negative judgment** (*n* = 26)^a^, ^b^	
Health neglect	13 (50.0)
Sexually transmitted disease	6 (23.1)
Drug abuse	6 (23.1)
Alcoholism	4 (15.4)
Lies	2 (7.7)
Patients who deny the diagnosis	2 (7.7)
**Do you have a negative judgment or annoyance regarding the patient's difficulty in understanding certain questions?** (*n* = 86)	
Yes	24 (27.9)
No	62 (72.1)

^a^ Variables with missing data.

^b^ Questions interpreted by Discourse of the Collective Subject (DCS).

## 4 Discussion

The present study highlights challenges and feelings experienced by dental students during their undergraduate course. STIs, negative diagnoses, and the need to deal with uncooperative patients were the main difficulties mentioned by the students in the care of their patients during clinical practice in oral medicine. The relationship between dentists and their patients can be complex and challenging. Both patient and professional are different people who carry unique life stories, and traumatic individual experiences may appear during the treatment [2, 6]. The dentists’ stigmas, prejudices, and judgments can interfere with their relationship with the patient and need to be better addressed throughout the undergraduate course [18].

Almost all the students included in this study reported feeling uncomfortable during the patient care. The main uncomfortable topics cited by the students during the patient care were non‐cooperative patients and communication problems. In fact, non‐cooperative patients or those who do not follow professional recommendations are an issue addressed by many healthcare professionals [19]. This problem reveals a communication gap between professionals and their patients. When patients feel they are not heard or understood by the professional, it can be interpreted as unprofessional behavior of the clinician, including insufficient empathy or inattention [19]. This gap in communication can be fulfilled with effective communication promoted by the professional, based on verbal, nonverbal, and listening channels focused on a patient‐centered approach [20].

About half of the students reported feeling uncomfortable when addressing a specific topic during patient anamnesis. The anamnesis process can be influenced by personality traits, social norms, and the method of data collection, which may lead to discomfort or inaccuracies [21]. The familiarization of the professional with the anamnesis process is crucial to an accurate diagnosis and a successful treatment, as it is a fundamental phase in clinical examination [22]. It is natural for students to feel awkward or uncomfortable dealing with patients at the beginning of their clinical lives. Therefore, it is essential that the educational institution offers adequate training in anamnesis to students, ensuring the formation of professionals familiar with this procedure.

The main uncomfortable topic reported by the dental students during the anamnesis was STIs, followed by drug abuse and sexual behavior. Patients with an STI diagnosis or people with substance use disorders (SUD) can receive negative stigma and are excluded in different proportions from social circles [10]. Social prejudice towards people diagnosed with sexually transmitted diseases is higher than towards people who have other illnesses [23]. As a result, patients with STIs feel ashamed to tell others about their diagnosis, trying to omit this personal information and hide the truth during the anamnesis to avoid external judgments [23]. This stigma also affects patients with SUD, who reported negative biases, prejudice, and discrimination in healthcare centers [24]. The embarrassment experienced by students in addressing stigmatized topics may have come from social learning stored in their unconscious values. To improve the anamnesis and care service provided by the dental students, these stigmas and prejudices must be addressed. The adoption of non‐stigmatizing language and anti‐bias education can reduce these unconscious stigmas and lead to a better relationship between professional and patient [25, 26].

In addition to the students' discomfort in approaching certain topics during anamnesis and patients’ care, our results also identified some adversities in dealing with patients’ emotional aspects. The students reported a bad feeling when the patient reports a negative life story or an emotionally traumatic life event. Despite these negative feelings, only a small proportion of students (26.7%) indicated that these emotions supposedly impact patient care. It is natural to feel empathy and solidarity when we are faced with patients in emotional or physical suffering [27, 28]. However, the health professional's exposure to the patient's high suffering contributes to emotional disorders (e.g., depression, burnout, high rates of suicide), and physical conditions, often leading healthcare professionals to abandon their careers [29, 30]. Resilience is also an important skill for healthcare professionals, being critical to ensuring their physical and mental well‐being [29, 30, 31]. Consequently, the emotional preparation of the students for their professional life should be one of the main topics to be addressed by the educational institutions during dental formation. Despite empathy and resilience were not properly evaluated in the present study, these abilities are relevant phenomena to be assessed among dental students in further investigations.

Our findings showed that the questions “Do you feel bad when the patient tells you a negative life story?” and “Do you feel discomfort when the patient tells a negative life story similar to yours?” were affirmatively answered by sixty‐five students (75.6%). Despite most of the students affirmatively answering both questions, 13 students (15.1%) reported emotional discomfort with a negative life story, but did not feel bad when this story was similar to theirs. With these answers, we can assume that the countertransference process is relatively common among dental students [32]. The human relationship, including the relationship between professional and patient, is based on exchanges that can awaken subjective feelings [33]. These transferences can be healthy or not, depending on the life experiences of each subject. In this way, several feelings that were previously unconscious may come to the surface during patient care [33]. This process is called countertransference, in which the clinician transfers his or her feelings to his or her client, often without realizing it when this happens [13, 34]. These feelings emerge from subjects or contexts that, for that specific person, represent something negative. In this way, to improve the students’ emotional resilience during patient care, it is necessary to work individually on each person's negative emotions according to their life story [35, 36].

This study has some limitations. Unfortunately, there is an absence of a validated questionnaire available in the literature that would respond to the objectives of the study. In this way, this study comprehends the application of a semi‐structured questionnaire elaborated by the authors instead of a previously validated instrument. Regarding the questions in the questionnaire, the interpretation of “discomfort” and “difficulty” may be influenced by the student's life experiences, personality, and emotional maturity. Therefore, the interpretation of the questions investigated in this research is dependent on the student's personal interpretation. Basically, the questionnaire questions assessed feelings and sensations, and not the student's behavior in relation to the patient. Therefore, there is a limited understanding of the possible behavioral consequences of the feelings triggered by the students towards the patient on the quality of care. Only one question explored the effects of the state of feeling on the care provided to the patient. In a future investigation, it would be possible to understand in greater depth how the feelings explored in the present study affect the care provided to the patient. Moreover, our study did not evaluate cognitive and affective components of empathy. A comprehensive understanding of the students’ empathic abilities would heighten the evaluation of the students’ difficulties and discomforts throughout patient care. Lastly, this study comprehends the evaluation of a specific population in only one research center. A major sample with a collaboration with different educational institutions would enhance the heterogeneity of our sample, which is influenced by the cultural and diversity aspects of the local students.

In conclusion, dental undergraduate students display evident difficulties in clinical practice with patients in the oral medicine clinic, especially in relation to some themes addressed during the anamnesis. Knowledge of these difficulties is essential to develop new learning and teaching strategies improving the students’ professional training during their dental undergraduate course. A more appropriate curriculum focused on the difficulties and feelings of students in caring for patients could better prepare them to face the challenges of clinical practice in their future professional careers Supporting Information.

## Conflicts of Interest

The authors declare no conflicts of interest.

## Supporting information




**Supporting File 1**: jdd70005‐sup‐0001‐SuppMat.docx
