# Supplementary material for: Difficulties and Feelings Experienced by Undergraduate Dental Students When Caring for Patients
Source: J Dent Educ. 2025 Aug 29;90(4):500–7. doi: 10.1002/jdd.70005 (PMC13077627; doi:10.1002/jdd.70005)
Supplement: Supplementary file 1 — Supporting File 1: jdd70005‐sup‐0001‐SuppMat.docx [file JDD-90-500-s001.docx]

**Questionnaire for evaluation of feelings and difficulties of undergraduate dentistry students in their relationship with patients during clinical practice**

This questionnaire aims to evaluate your feelings and behaviors regarding patient care during clinical practice. The results will be analyzed collectively to identify the challenges faced by graduate students in clinician-patient interactions. We kindly ask you to answer the questions as honestly as possible.

**1. SOCIODEMOGRAPHIC AND BIOBEHAVIORAL VARIABLES**

1.1 Gender: _______________________________ 1.2 Age: ______________________________________

1.3 Religion: _____________________________________________________________________________

1.4 How often do you practice your religion?

( ) Weekly ( ) Monthly ( ) Occasionally ( ) Religion is not part of my routine.

1.5 What is your family’s socioeconomic status?

( ) High-income ( ) Middle-income ( ) Low-income

1.6 Are you currently undergoing treatment for any disease? If yes, please identify:

__________________________________________________________________________________________________________________________________________________

1.7 Do you smoke cigarettes or other tobacco products (Cigarettes, Cigars/Pipes, E-cigarettes/Vaping, Hookah? ( ) Yes ( ) No

If yes, please indicate:

Type of cigarettes or other tobacco products: ____________________________________________________

What is the frequency of use of this type of product? ( ) Daily ( ) Weekly ( ) Monthly.

Indicate another type of cigarettes or other tobacco products that you also consume: ______________________

What is the frequency of use of this type of product? ( ) Daily ( ) Weekly ( ) Monthly.

1.8 Do you consume alcoholic beverages? ( ) Yes ( ) No

If yes, please indicate:

Type of alcoholic beverages: _________________________________________________________________

What is the frequency of use of this type of product? ( ) Daily ( ) Weekly ( ) Monthly.

Indicate another type of alcoholic beverages that you also consume: __________________________________

What is the frequency of use of this type of product? ( ) Daily ( ) Weekly ( ) Monthly.

**2. FEELINGS AND BEHAVIOR DURING PATIENT CARE**

2.1 In the context of clinical anamnesis, do you feel uncomfortable asking the patient any question?

( ) Yes ( ) No

If yes, indicate which question and the reason why it makes you uncomfortable.

__________________________________________________________________________________________________________________________________________________

_________________________________________________________________________

2.2 Do you have difficulty discussing any specific topics with patients? ( ) Yes ( ) No

If yes, indicate which topic and the reason.

__________________________________________________________________________________________________________________________________________________

_________________________________________________________________________

2.3 During the whole patients’ care, does any topic make you uncomfortable?( ) Yes ( ) No

If yes, indicate which topic and the reason.

__________________________________________________________________________________________________________________________________________________

_________________________________________________________________________

2.5 Do you feel bad when the patient tells you a negative life story? ( ) Yes ( ) No

2.6 Do you feel bad when the patient reports traumatic emotional events that occurred in their life?

( ) Yes ( ) No

2.7 Do you feel discomfort when the patient tells a negative life story similar to yours?

( ) Yes ( ) No

2.8 When the patient tells you a negative life story, does this impact on the care you provided?"

( ) Yes, it negatively impacts the care provided. ( ) No, it does not impact the care provided

2.9 Have you had a negative judgment towards patients when they answered some question in the interview?

( ) Yes ( ) No

If yes, indicate which question topic whose answer generated the negative judgment.

__________________________________________________________________________________________________________________________________________________

_________________________________________________________________________

2.10 Do you have a negative judgment or annoyance regarding the patient's difficulty in understanding certain questions? ( ) Yes ( ) No
